# Understanding the role of family functioning, dietary adherence, and culture on glycemic control among adults with type 2 diabetes: A mediation and moderation analysis

**DOI:** 10.1371/journal.pone.0320235

**Published:** 2025-04-01

**Authors:** Halimatou Alaofè, Abidemi Okechukwu, Waliou Amoussa-Hounkpatin, Iman A. Hakim, Carmelle Mizéhoun-Adissoda, Jules Gninkoun, Edward John Bedrick, John Ehiri

**Affiliations:** 1 Department of Health Promotion Sciences, Mel and Enid Zuckerman College of Public Health, The University of Arizona, Tucson, Arizona, United States of America; 2 School of Nutrition and Food Science and Technology, Faculty of Agricultural Sciences of the University of Abomey-Calavi (FSA-UAC) Campus d’ Abomey-Calavi, Calavi, Benin; 3 School of Nutrition and Dietetics, Faculty of Health Sciences, University of Abomey-Calavi, Calavi, Benin; 4 Faculty of Health Sciences, University of Abomey Calavi, Calavi, Benin; 5 Department of Epidemiology and Biostatistics, Mel and Enid Zuckerman College of Public Health, The University of Arizona, Tucson, Arizona, United States of America; Instituto Nacional de Cardiologia Ignacio Chavez, Mexico

## Abstract

**Background:**

Families and cultural contexts can impact dietary adherence and glycemic control of type 2 diabetes (T2D). Yet little is known about these relationships in Africa, where poor dietary adherence and glycemic control are prevalent. To address this gap, this study investigated whether dietary adherence mediates family functioning and glycemic control among T2D adults in Benin, West Africa. We also explored whether cultural identity affected the association between family functioning and dietary adherence.

**Methods:**

A cross-sectional study of 512 T2D patients from six health centers was conducted to assess: 1) family functioning with the 12-item McMaster Family Assessment Device–General Functioning Subscale (FAD-GF); 2) dietary adherence via the Perceived Dietary Adherence Questionnaire (PDAQ); and 3) cultural identity with the 12-item Multigroup Ethnic Identity Measure (MEIM). The three-month glycemic control was determined with Glycated Hemoglobin - HbA1c. Mediation and moderation analyses were conducted using Stata’s structural equation model (SEM).

**Results:**

Healthy family functioning, good dietary adherence and good glycemic control rates were 56.8%, 33%, and 30.5% respectively. Path analysis showed that healthy family functioning was significantly associated with lower HbA1c levels (-0.34, 95% CI: [-0.72, -0.03]), and there was a significant indirect effect via greater dietary adherence (-0.12, 95% CI: [-0.22, -0.01]). However, cultural identity did not significantly impact the relationship between family functioning and dietary adherence.

**Conclusions:**

Our study revealed that family functioning, adherence to dietary recommendations, and glycemic control are interconnected in adults with T2D. Interventions should target modifiable factors like dietary adherence and address relevant risk and resilience sources to improve glycemic control in urban African families.

## Introduction

The global burden of Type 2 diabetes (T2D) is escalating, particularly in resource-limited settings, leading to a double burden of communicable and chronic diseases [1]. Over the last two decades, newly diagnosed T2D cases have tripled, and projections indicate that by 2030, 1 in 10 adults will have T2D [2]. This surge in diabetes incidence imposes a substantial financial strain on individuals, societies, and countries, with treatment costs totaling $1.3 trillion annually. In addition to its economic burden, T2D also presents a challenge to mental health; about 40% of people with T2D experience decreased psychological well-being [3].

The sub-Saharan Africa (SSA) region is no exception, as it is expected to experience a significant increase in T2D prevalence, with a projected growth of 143% by 2030 [4,5]. Compliance with medication and dietary guidelines is crucial for achieving positive health outcomes for T2D patients. However, despite its significant health and social implications, poor adherence to dietary recommendations - a fundamental aspect of T2D management - is often overlooked [6,7]. In addition, evidence suggests that individuals with T2D in SSA face unique challenges, such as increased risk for low adherence and poor glycemic control, worsened by poverty, limited social support, and the challenges of urban living within households and family settings [8,9]. Therefore, it is essential to understand SSA-specific factors contributing to this epidemic to address its rising prevalence. Interventions tailored to cultural and contextual settings have the potential to notably improve T2D prevention, treatment management, and health outcomes, offering hope in this crisis [10].

The literature suggests that T2D is a family disease, with evidence showing that T2D management and outcomes are strongly interconnected with family health and functioning [11,12]. Unhealthy family functioning is associated with higher glycated hemoglobin - HbA1c [13], whereas people with T2D from well-functioning families report better diabetes outcomes [14,15]. Family functioning encompasses various aspects of family dynamics, such as communication, conflict resolution, cohesion, and structure, all of which play a crucial role in influencing T2D outcomes [16]. Additionally, poor adherence to dietary recommendations, essential for managing blood sugar levels [17,18], correlates with inadequate family support and functioning [19,20]. Given the critical role of dietary adherence in glycemic control, we hypothesized that dietary adherence acts as a mediator between family functioning and glycemic control. Understanding these relationships can provide valuable insights for healthcare professionals, researchers, and policymakers, guiding efforts to leverage social and family functioning to enhance dietary adherence and glycemic control for T2D patients.

A significant portion of the literature advocating “culture as a treatment” focuses on dietary aspects of SSA. This emphasis is due to the region’s diverse cultural variations that influence diabetes-related behaviors, making them crucial for addressing the diabetes epidemic [21,22]. Previous studies have suggested that cultural practices, such as food restrictions in Islam, can hinder adherence to dietary guidelines. However, engaging in traditional societal customs can also prevent unhealthy eating habits [23]. For instance, Onyishi et al. [24] found a positive correlation between spiritual beliefs and effective diabetes management. Qualitative studies have highlighted the importance of spirituality, traditional customs, and cultural identity in coping with and managing diabetes [25,26]. Nevertheless, there is a lack of research examining the impact of cultural identity on family dynamics, dietary adherence, and blood sugar control. Given the pivotal role of food habits in preventing and managing T2D and the cultural influences prevalent in SSA, it is crucial to examine how cultural context, including cultural identity, affects the relationship between family dynamics, dietary adherence, and blood sugar management.

**Fig 1:**
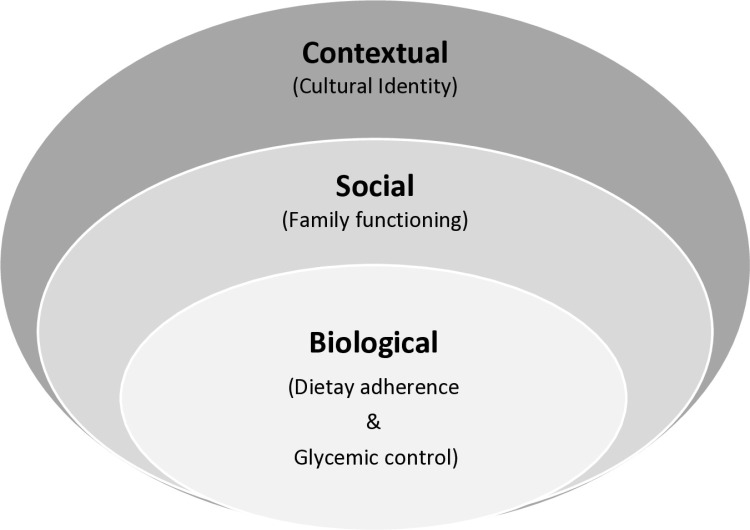
The developmental psychopathology framework.

The biopsychosocial and contextual paradigms are an essential theoretical framework for understanding the relationships between family functioning, dietary adherence, and glycemic control (Fig 1) [27]. Biological, psychological, social, and contextual factors, such as glycemic control and dietary adherence, are linked to various social and family factors [27, 28]. The biological dimension of this model emphasizes the importance of studying the link between dietary adherence and glycemic control, given the role of diet in the development and management of T2D [29]. Social factors, such as family functioning, impact glycemic control and dietary adherence. However, little is known about the effects of family functioning on dietary behavior and health outcomes in SSA [30,31]. The contextual component of this model highlights the significance of cultural factors in family functioning, dietary adherence, and glycemic control. Understanding cultural influences is critical, given African societies and families’ diverse cultural values and beliefs. Therefore, this study aims to investigate whether dietary adherence mediates the link between family functioning and glycemic control among T2D adults in a research sample from the Republic of Benin, West Africa. Furthermore, the study examines the potential moderating role of cultural identity in the relationship between family functioning and dietary adherence.

## Materials and methods

### Study design, settings, and participants

This cross-sectional study was conducted between October and December 2023 in six secondary care centers in Parakou and Cotonou, the largest northern and southern Benin cities, respectively. In recent years, both cities have experienced growth in commerce, as is evident in establishing multiple banks, hotels, schools, markets, and fast-food restaurants, leading to changes in dietary and social lifestyles [32, 33]. It is hypothesized that these factors contributed to the increased prevalence of diabetes, from 4.9% to 19.4% in Cotonou and from 4.6% to 21.6% in Parakou between 2008 and 2015 [34, 35]. The secondary health centers involved in the study were the Bank of Insulin, NGO Diabetes Benin of Parakou, and four private clinics (BONI of Akpakpa, Saint Pio of Cotonou, EndoDiab21, and Biosso). The Bank of Insulin in Cotonou and NGO Diabetes Benin in Parakou are national reference centers for screening and managing diabetes.

To participate in the study, patients must be patients with Type 2 Diabetes (T2D) receiving care in the outpatient department of the diabetes unit in the six health centers. To be eligible, they must meet the following criteria: (1) be between 35 and 65 years old, (2) have a documented diagnosis of T2D, (3) self-identify as Beninese, (4) have been living with T2D for a year or more, and (5) be willing to provide informed consent to participate. However, patients with severe health conditions that hinder their ability to complete questionnaires or participate in biological (e.g., blood draws) and anthropometric (e.g., height and weight) assessments are excluded from the study.

### Sample size determination and sampling techniques

Using prior analyses [17,20] and based on Fritz and Mackinnon’s suggestion [36,37], a sample size of 444 is needed for 80% power. To account for a potential 10% non-response rate to the interview and/or missed data, we approached 512 patients for the final study. The final sample size was then proportionally divided among the selected health centers to obtain a representative sample from each center. All eligible T2D patients during the data collection periods were approached until the required sample size was achieved using sequential sampling. Ultimately, we enrolled 244, 102, 43, 29, 47, and 47 eligible patients from the Bank of Insulin, NGO Diabetes Benin, clinic BONI, clinic Saint Pio, clinic EndoDiab21, and clinic Biosso, respectively, based on the number of T2D patients in each participating center.

### Data collection instruments and procedures

As part of the study protocol, we collected information regarding various aspects through a pretested interviewer-administered questionnaire that included validated data collection instruments. The information collected consisted of the following: (1) family and social characteristics, (2) family functioning evaluated using the 12-item McMaster Family Assessment Device–General Functioning Subscale (FAD-GF), (3) family support assessed with the Multidimensional Scale of Perceived Social Support (MSPSS), (4) dietary adherence measured with the Perceived Dietary Adherence Questionnaire (PDAQ), (5) cultural identity analyzed using the 12-item Multigroup Ethnic Identity Measure (MEIM), and (6) physical measurements such as height, weight, waist and hip circumference, and laboratory examination of Glycated Hemoglobin – HbA1c.

The questionnaire was prepared in English and translated into French (the official language) by professionals fluent in both languages. Then, it was translated back into English to ensure accuracy and consistency. The research team trained data collectors for four days on data collection procedures and ethical issues to ensure reliability. The questionnaire was piloted with fifteen T2D patients in another hospital to assess its content suitability and flow. Trained lab technicians collected all anthropometric measures and capillary blood samples for HbA1c using sterile techniques according to York University’s biosafety and ethics requirements.

#### Family and social characteristics.

In the socio-demographics section of the questionnaire, the participants were asked to report their age, gender, marital status, ethnicity, religion, highest level of education, duration of diabetes, consumption of tobacco and alcoholic beverages, and sources of dietary advice. In the family characteristics section, they were asked to report the type of family they belong to (monogamy, polygamy, or single-parent family), work status, household size, and monthly income (MI).

#### Family functioning.

Family functioning was assessed using the 12-item McMaster Family Assessment Device–General Functioning Subscale (FAD-GF), widely validated across studies [38, 39]. The GF-12 covers the following aspects of family functioning: problem-solving (one item), communication (four items), roles (two items), affective responsiveness (one item), affective involvement (three items), and behavioral control (one item). The 12 items include six positive and six negative statements describing healthy and unhealthy family functioning. Each item is scored on a 4-point Likert scale: Strongly agree = 1; Agree = 2; Disagree = 3; Strongly disagree = 4. The final score was obtained by summing up the items’ scores and dividing them by 12. The final score of ≤  2.0 meant healthy family functioning, while a score of > 2.0 indicated unhealthy family functioning.

#### Social support.

The level of social support was assessed using the Multidimensional Scale of Perceived Social Support (MSPSS) [40,41]. This scale assesses the perceived adequacy of social support from three sources: family, friends, and significant others. Respondents were asked to rate their agreement with 12 statements on a 5-point Likert scale (0 =  strongly disagree, 5 =  strongly agree). To calculate a mean score for each subscale, we summed up the respective items and divided them by the number of items. For instance, we added items 6, 7, 9, and 12 to calculate a mean score for the friend’s subscale and then divided the sum by 4. Finally, we defined response descriptors: a mean score of 1 to 2.9 indicated low support, 3 to 5 indicated moderate support, and 5.1 to 7 indicated high support.

#### Dietary adherence.

The Perceived Dietary Adherence Questionnaire (PDAQ) was used to measure how well participants followed diabetes dietary recommendations. The questionnaire consists of nine questions based on the WHO guidelines and the Benin Food Guide-BFG [42–44]. The questions cover various aspects of a healthy diet, such as overall adherence to a dietary plan, recommended servings of fruits and vegetables, consumption of low glycemic index carbohydrate-containing foods, high-sugar foods, high-fiber foods, n-3 fatty acids, healthy (monounsaturated) oils, and high-fat foods. One question addresses appropriate carbohydrate spacing. Participants answered the questions using a seven-point Likert scale (0-7), which measured how often they followed a healthy diet over the past seven days. Higher scores indicate better adherence to a healthy diet, except for questions 4 and 9, which reflect unhealthy food choices (high in sugar or fat). For these questions, higher scores indicate lower adherence. Therefore, the scores for these questions were inverted when calculating the total PDAQ score. Although the questionnaire is based on a weekly timeframe, it is expected to reflect participants’ usual dietary patterns, as most people tend to consume similar foods from week to week. Participants were considered to have good dietary adherence if they followed a healthy diet at least four days a week.

#### Cultural identity.

We used the Multigroup Ethnic Identity Measure (MEIM) questionnaire to evaluate ethnic identity. This questionnaire comprises 12 items and has been widely used in various studies. It has also consistently demonstrated high reliability across different age ranges and ethnic groups [45]. The questionnaire contains two factors, namely ethnic identity search and affirmation, belonging, and commitment. To calculate the overall score, we computed the mean of the 12 items rated on a four-point Likert scale from 1 (strongly disagree) to 4 (strongly agree). A high score indicates that the individual has achieved ethnic identity, showing they have explored and committed to it. Conversely, a low score indicates a diffuse ethnic identity, which means they lack exploration and commitment.

#### Anthropometric & glycemic control measurements.

The study included measurements of various physical characteristics of the participants, such as weight, height, waist, and hip circumference. Weight was measured in kilograms to the nearest 0.1 kg with a standard weighing scale (Hana model, China) while ensuring the participants wore light clothing and no shoes. Height was measured with a stadiometer to the nearest 0.1 cm. The body mass index (BMI) of each participant was calculated using the formula: BMI =  weight (kg)/Height2 (m2). We used WHO classifications for adult BMI: underweight (under 18.5 kg/m2), normal weight (18.5 to 24.9), overweight (25 to 29.9), and obese (30 or more) [46]. Waist circumference was measured with the participant standing upright, and hip circumference was taken to calculate the waist-hip ratio. A waist-hip ratio above 0.90 for males and 0.85 for females indicates abdominal obesity [47].

We assessed glycemic control by testing HbA1c levels using point-of-care fingerstick capillary blood testing. HbA1c was chosen as the primary blood marker because it is a simple and minimally invasive measure that does not require fasting, allowing for flexible testing. Additionally, it has been included in the diagnostic criteria for Type 2 diabetes by the World Health Organization (WHO) and ADA [48, 49]. In our study, we used the HemoCue® HbA1c 501 System for HbA1c tests, and a level greater than 7% was considered indicative of poor glycemic control [50].

### Ethical considerations

The study protocol initially received approval from the Institutional Review Boards of the University of Arizona and the National Ethics Committee for Health Research (CNRES) of Benin. Subsequently, permission was obtained from the participating institutions to proceed with the study. After the study objectives were explained to participants, they were asked to complete and sign their informed and written consent forms to participate in the study. Confidentiality was strictly maintained, and the study was conducted in accordance with Helsinki legislation, ensuring the data was anonymized thoroughly.

### Data analysis

All statistical analyses were performed using Stata 18 (StataCorp, 2023). We first analyzed descriptive quantitative data, calculating means and standard deviations for continuous variables and counts and percentages for categorical variables. We then conducted mediation and moderation analyses using the structural equation model (SEM) after confirming that the necessary assumptions for mediation analysis were met. These assumptions include linearity, normality, homogeneity of error variance, independence of errors, correct model specification, and the absence of spurious outliers [51–53].. All models were controlled for age, sex, education, duration of diabetes diagnosis, work status, marital status, BMI, health centers, and social support, as these confounding variables are related to dietary adherence and glycemic control [54–56]. Family functioning was coded as a binary variable while dietary adherence and glycemic control were continuous variables. Although we have very few missing data (only two values for BMI), we conducted sensitivity analysis that shows our primary findings remain robust even under scenarios where missing data is assumed to be more systematically related to key study variables, providing confidence in the overall conclusions despite the low missing data rate.

**Fig 2:**
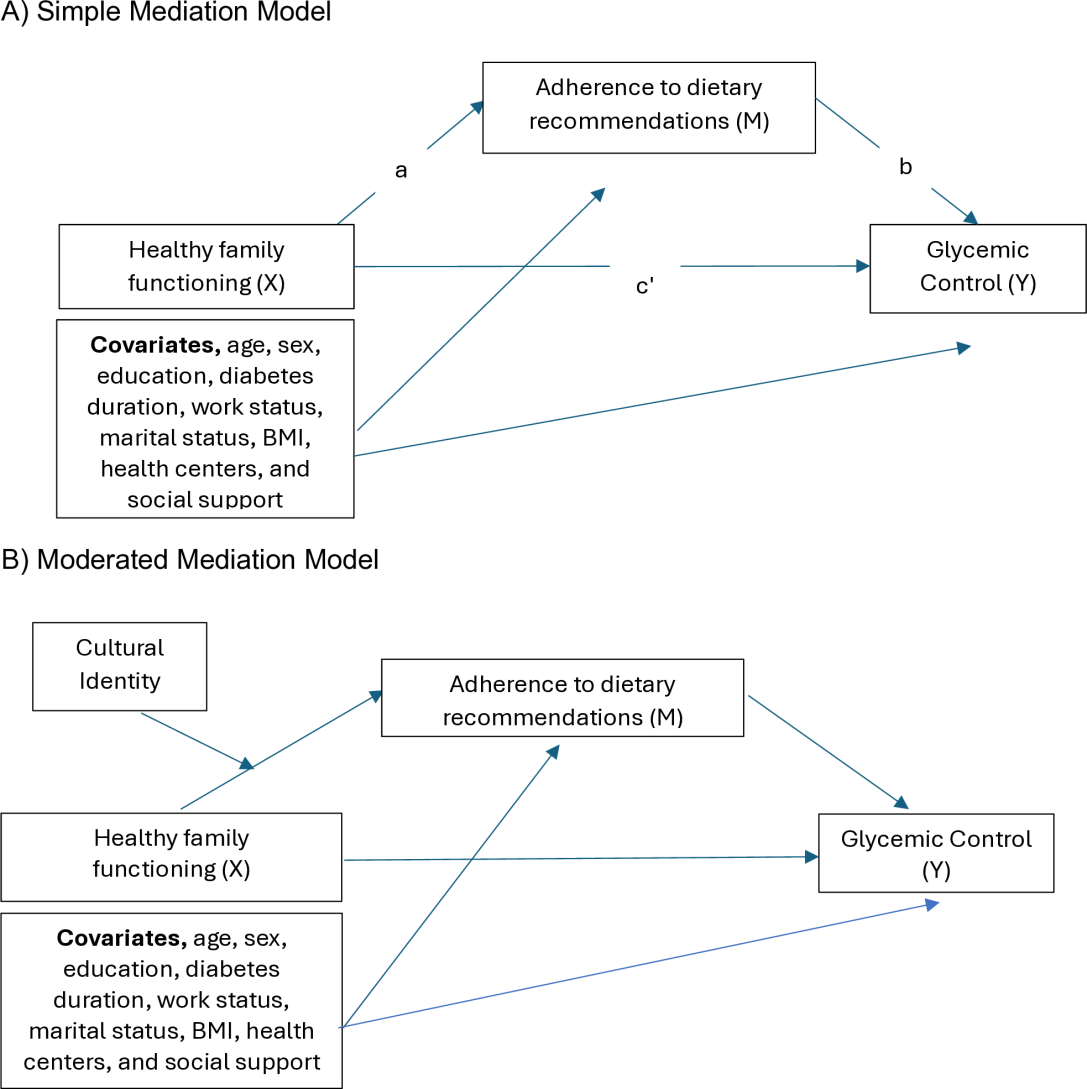
Conceptual diagrams to assess how family functioning, dietary adherence, and culture affect glycemic control through mediation and moderation.

We tested a simple mediation path model (Fig 2a), examining whether the indirect effect (a * b) of family functioning on glycemic control through dietary adherence was statistically significant. We also tested a moderated mediation model, examining whether cultural identity moderated the indirect effects of healthy family functioning on dietary adherence (Fig 2b). We used a bootstrapping procedure with 5,000 replications to test the statistical significance of indirect and moderated mediation effects. Bootstrap 95% confidence intervals are reported [57], and we set a significance level of 0.05. Model fit for mediation models was evaluated using a combination of the comparative fit index (CFI), Tucker–Lewis index (TLI), root-mean-square error of approximation (RMSEA), and standardized root-mean-square residual (SRMR), following guidelines from the literature [58,59]. We considered the models acceptable if the CFI and TLI were greater than 0.95, the RMSEA and SRMR was less than 0.08, and the SRMR was less than 0.05.. Standardized coefficients, also known as effect sizes are reported. These coefficients are standardized relative to the dependent variable Y and the independent variable X, indicating the change in Y measured in standard deviation units when X changes by one standard deviation. We interpret these coefficients using the following conventions: values of 0.20, 0.50, and 0.80 correspond to small, medium, and large effects, respectively. [60].

(A) This Directed Acyclic Graph (DAG) diagram illustrates the simple mediation models used to examine whether healthy family functioning indirectly affects glycemic control through dietary adherence. The indirect effect is estimated as the product of paths a and b (a * b). (B) This Directed Acyclic Graph (DAG) illustrates the moderated mediation models. We examined whether Benin cultural identity influences the indirect effects of healthy family functioning on dietary adherence.

## Results

### Socio-demographic and clinical characteristics of study participants

Table 1 presents the characteristics of the study participants, including their socio-demographics, physical measurements, lifestyle habits, and glycemic profile. The participants were aged 35 to 65 years, with the majority (80.9%) falling between 41 and 60 years old. Most of the 512 participants were married (75.2%), female (60.9%), and had completed secondary or university education (60.2%). In terms of family structure, most of them lived in nuclear families (73%), were engaged in independent work (61.7%), and had low incomes (<$166; 59.6%). Concerning social support, friends’ support was low, with an average score of only 3.83 ± 1.58. As for physical measurements and lifestyle habits, 73.3% were overweight, with 61.6% having a moderate to low waist-to-hip ratio. Although 52.1% reported alcohol consumption, most (44.7%) reported occasional drinking. Additionally, while 87.1% received nutrition counseling, only 7.6% received advice from a dietitian or nutritionist. Finally, only 30.5% of the participants had an HbA1c level of less than 7%.

**Table 1:** Socio-demographic and clinical characteristics of study participants (n = 512).

Variables	Frequency, n (%)
**Socio-demographics**	
Age (mean ± SD) years	52.18 ± 7.77
≤40	52 (10.16)
41-60	414 (80.86)
>60	46 (8.98)
Sex	
Female	312 (60.94)
Male	200 (39.06)
Marital status	
Married	385 (75.20)
Single	64 (12.50)
Widow/widower	63 (12.30)
Ethnicity	
Fon	214 (41.80)
Yoruba	98 (19.15)
Adja	32 (6.25)
Bariba	52 (10.16)
Others^a^	116 (22.64)
Religion	
Muslim	130 (25.39)
Christian	250 (48.83)
Others^b^	132 (25.78)
Education	
No formal education	89 (17.38)
Primary	115 (22.46)
Secondary	201 (39.26)
University	107 (20.90)
Diabetes duration (mean ± SD) years	7.88 ± 6.85
Type of family	
Monogamy	374(73.05)
Polygamy	138(26.95)
Work status	
Unemployed	40(7.81)
Government employment	55(10.74)
Non-governmental employment	60(11.72)
Independent worker	316(61.72)
Retired	41(8.01)
Family size	
≤5	344(67.19)
>5	168(32.81)
Monthly income	
<$166	305(59.57)
$166-$332	134(26.17)
>$332	73(14.26)
Social Support (mean ± SD)	
Family subscale	5.33 ± 1.39
Friends Subscale	3.83 ± 1.58
Significant Other Subscale	5.57 ± 1.41
Total Scale	4.91 ± 1.09
**Anthropometrics and lifestyle habits**	
BMI (mean ± SD) kg/m^2^ (n = 510)	29.11 ± 6.30
Underweight	11(2.17)
Normal	125 (24.51)
Overweight	179 (35.10)
Obese	195 (38.24)
Waist/Hip ratio (mean ± SD) cm	0.89 ± 0.10
Low	209 (40.98)
Moderate	105 (20.59)
High	196 (38.43)
Lifestyle	
Smoking habit	18 (3.52)
Alcoholic drink habit	267 (52.15)
Source of diet advice	446 (87.11)
Dietitian	34 (7.62)
Other^c^	412 (97.38)
**Glycemic profile**	
Hb A1c (mean ± SD)	8.36 ± 2.11
≤7%	156 (30.47)
>7%	356 (69.53)

^a^Dendi, Goun, peulh & Haoussa.

^b^Céleste, Protestant/Evangelist, Eckankar.

^c^Endocrinologist, nurse, general physician, family and friends

### Family functioning, dietary adherence and cultural identity among study participants

Table 2 presents detailed information about family functioning, dietary adherence, and cultural identity among the study participants. The mean family function score was 1.98 ± 0.45, and 53.3% of participants reported healthy family functioning. Regarding dietary adherence, participants consumed a significant amount of high-fat foods, such as high-fat dairy products and fried foods, with an average score of 4.98. They also had a notable intake of n-3 FA foods, such as fish or other foods rich in omega-3 fats, with an average score of 4.43. However, their consumption of low GI, high-fiber foods, adherence to a healthy eating plan, and spacing out carbohydrate intake were low, with average scores of 2.25, 2.31, 2.76, and 2.87, respectively, for fewer than four days within a week. Their intake of healthy oils, high-sugar foods, and fruits & vegetables was also low, with average scores of 1.36, 1.42, and 1.85 within the last seven days, respectively. According to the PDAQ, most participants (67%) had poor dietary adherence. The results also indicated a strong cultural identity, with a mean score of 3.02, and a greater commitment to ethnic identity than a search for it (3.16 vs. 2.82). Detailed information about family functioning, dietary adherence, and cultural identity among the study participants is presented in (S1-S3 Tables in the S1 File).

**Table 2:** Family functioning, dietary adherence and cultural identity among study participants.

Outcomes variables	Mean ± SD	Frequency, n(%)
**Family functioning**		
FAD-GF family class (total score)	1.99 ± 0.45	
Healthy family functioning ( ≤ 2.0)		296(56.84)
Unhealthy family functioning ( > 2.0)		221(43.16)
**Dietary adherence**		
PDAQ (Total score)	3.26 ± 0.63	
Poor dietary adherence(<4)		343 (66.99)
Good dietary adherence (≥4)		169(33.01)
**Cultural identity**		
Exploration	2.82 ± 0.53	
Commitment	3.16 ± 0.53	
MEIM (Total score)	3.02 ± 0.49	

FAD-GF = McMaster Family Assessment Device–General Functioning Subscale; PDAQ = Perceived Dietary Adherence Questionnaire; MEIM = Multigroup Ethnic Identity Measure.

### Effect of dietary adherence and cultural identity on family functioning and glycemic control

As shown in Table 3, healthy family functioning was significantly associated with lower HbA1c levels (-0.34, 95% CI: [-0.72, -0.04]), and there was a significant indirect effect via greater dietary adherence (-0.12, 95% CI: [-0.22, -0.01]). In other words, healthy family functioning was associated with greater dietary adherence (1.57, 95% CI: [0.47, 2.67]), which in turn was associated with lower HbA1c levels (-0.08, 95% CI: [-0.11, -0.04]). In particular, the total effect for family functioning (-0.46) is significant with a z of -2.34 (95% CI: [-0.84, -0.07]). The direct effect for family functioning is -0.34 which, while still significant (z =  -1.73, 95% CI: [-0.72, -0.04]), is much smaller than the total effect. The indirect effect of family functioning that passes through dietary adherence is -0.12 and statistically significant. As for the effects sizes, the proportion of the total effect that is mediated is almost 0.30 which is in the small range. The ratio of the indirect effect to the direct effect is about 0.35 or almost 1/3 the size of the direct effect. And finally, the total effect is about 1.35 times the direct effect. Model fit was acceptable (CFIs ≥ 0.95, TLIs ≥ 0.90, RMSEAs ≤ 0.08, SRMR < 0.05).

**Table 3:** Indirect effects of dietary adherence on family functioning and glycemic control* .

	Path c’ (X → Y)	Path ^a^ (X → M)	Path ^b^ (M → Y)	Indirect effects (a * b)
Model Coefficient [Bootstrap 95% CI]	-0.34 [-0.72, -0.04]	1.57 [0.47, 2.67]	-0.08 [-0.11, -0.04]	-0.12 [-0.22, -0.01]

*95% CI = 95% confidence interval. X =  independent variable = Healthy family functioning. M =  mediator=Dietary adherence. Y =  dependent variable = Glycemic control (HbA1c).

As shown in Fig 2b, we tested whether the observed indirect effects of family functioning on glycemic control via dietary adherence were moderated by cultural identity. We did not find any significant moderated mediation effects, suggesting that indirect effects did not differ significantly at different levels of cultural identity (-0.09; 95% CI: [-0.25, -0.05]).

All models controlled for age, sex, health centers, education, BMI, social support, diabetes duration, occupation, and marital status. Model fit was acceptable (CFIs ≥ 0.95, TLIs ≥ 0.90, RMSEAs ≤ 0.08, SRMR < 0.05)

## Discussion

This study aimed to fill gaps in the existing literature by exploring the role of family functioning and culture in behavior and health outcomes, such as dietary adherence and glycemic control, in SSA. Our study found a strong connection between family dynamics and glycemic control, with dietary adherence being a key mediator in this relationship. These results align with previous findings that showed a positive link between healthy family functioning and better outcomes for T2D patients, while unhealthy family functioning can hurt diabetic care, leading to poor dietary adherence, glycemic control, and other adverse outcomes [13,15,51]. This suggests that healthcare providers treating T2D patients from unhealthy family environments may mistakenly attribute a lack of progress in dietary adherence to therapeutic failure. Instead, they should recognize the impact of family functionality in diabetic care and provide psychosocial support to improve dietary adherence and blood glucose levels [61]. Moreover, it is vital to educate families with diabetic members about this connection and encourage their active involvement in the patient’s care, especially in resource-challenged settings where options for optimal diabetic care and healthy living are limited.

We found that healthy family functioning is significantly related to dietary adherence. This supports previous research, which suggests that improving family functioning in diabetic patients can help them stick to their diet, while poor family functioning can result in reduced self-care activities, such as dietary adherence [19, 20]. It is, therefore, not enough to provide diet plans alone. We should regularly evaluate family functioning as part of standard diabetic care. Diabetic patients with family issues should receive appropriate family-oriented interventions to improve family functionality. Additionally, due to the various food restrictions, such as cutting out certain enjoyable foods, it is understandable that only about 8% of patients seek advice from dietitians. Thus, diabetic patients should learn about healthy diets from a dietitian or treating physician and receive support from their family members. It could be beneficial to discuss the challenges of sticking to a diet not only with a diabetes specialist or a dietitian but also with family members [62]. Individuals with access to various forms of social support, such as emotional, informative, instrumental, and others, are better equipped to handle stress related to the disease. Adequate social support can impact the development of a positive and proactive attitude towards the disease and the ongoing treatment process [63].

Previous work has emphasized cultural identity’s important protective role in improving dietary adherence [64,65]. However, we did not find that cultural identity moderated associations between family functioning, dietary adherence, and glycemic control. This may be due to the African population’s complex, dynamic, and multi-dimensional nature of cultural identity [21]. Null results may also be explained by the MEIM scale not capturing the most salient cultural identity factors among T2D adult patients. For example, prior work with this scale discussed aspects of identity that require additional examination, such as the role of engagement in specific cultural activities or the role of globalization in cultural identity [66]. Especially in Africa, distinct social groups have different habits and behaviors, which specifically present the needs of each group, their cultural conditions, their experiences, and their history, among other aspects, thus establishing a particular distinction that can be recognized [67,68]. Further research and psychometric evaluations may enhance our understanding of optimal measurement scales for cultural identity in complex African life.

### Limitations & strengths

The study has some limitations. Firstly, causal inferences cannot be made because the mediation and moderated mediation models were based on cross-sectional data that did not establish temporality between family functioning and glycemic control. Future research should take a longitudinal approach to explore the relationship between family functioning, dietary adherence, and glycemic control, especially considering the cultural context. Secondly, while participants were encouraged to be honest and accurate, there is potential for reporting bias during the questionnaire and interview process. Third, this facility-based cross-sectional survey is inherently prone to selection bias and may not represent the general population. Information about T2D patients who do not use these facilities may be missing. However, this selection bias was minimized by strategically selecting clinics that represent the geographic and socio-economic diversity of the country (north and south). Finally, discrepancies in quality and capabilities between these health facilities, especially in providing and communicating appropriate dietary recommendations, could affect the findings, making comparisons between people with T2D challenging.

Despite these limitations, this study provides valuable data with relevant implications for diabetic care. One strength of the study is its use of HbA1c values to define glycemic control levels, which have the lowest intra-individual variation compared to fasting plasma glucose and oral glucose tolerance tests [69]. Additionally, the study did not rely on self-reported weight, height, waist, and hip circumference measurements, which were often underreported in several studies [70,71]. Furthermore, the study offers evidence of the growing importance of family functionality in glycemic control and its potential impact on developing effective interventions to improve blood glucose control. T2D is a multi-dimensional disease that impacts family and community health in SSA. Diabetic patients with unhealthy family dynamics are often encountered in the care setting in the study area (43.1% in the present study), and family dynamics play a vital role in factors that maintain optimum health in T2D management, especially in ambulatory care environments. Regular evaluation of family functioning can provide clinicians with additional information on diabetic patients at risk of low dietary adherence and poor glycemic control.

## Conclusions

This study critically examines the pathway between family functioning and glycemic control among Type 2 diabetes (T2D) patients and how dietary adherence and cultural context contribute to this association. The analysis indicated that healthy family functioning was linked to better glycemic control through increased dietary adherence. The results suggest that family-based interventions should be tailored to balance family support while considering potential barriers to dietary adherence. Consequently, addressing dietary adherence with families may enhance the overall well-being of T2D patients. Furthermore, further research should explore how cultural identity can strengthen family unity and resilience, ultimately decreasing the risk of low glycemic control in Africa.

## Supporting information

S1 File Supporting information. (ZIP)
