# Supplementary material for: Understanding the role of family functioning, dietary adherence, and culture on glycemic control among adults with type 2 diabetes: A mediation and moderation analysis
Source: PLoS One. 2025 Apr 1;20(4):e0320235. doi: 10.1371/journal.pone.0320235 (PMC11960932; doi:10.1371/journal.pone.0320235)
Supplement: S1 File — (ZIP) [file pone.0320235.s001.zip › Supporting Information/Supplement Tables.docx]

S1 Table. Family functioning among study participants.

| **Outcomes variables** | **Mean ± SD** |
| --- | --- |
| Planning family activities is difficult because we don't understand each other.  In times of crisis, we can turn to each other for help.  We can't talk to each other about the sadness we feel.  Individuals are accepted for who they are.  We avoid discussing our fears and concerns.  We can express our feelings to each other.  There are a lot of bad feelings in the family.  We feel accepted for who we are.  Making decisions is a problem for our family.  We are able to decide solutions to problems.  We don't get along well.  We trust each other. | 2.61 ± 0.87  1.85 ± 0.69  2.75 ± 0.81  1.80 ± 0.61  2.83 ± 0.78  1.88 ± 0.68  2.91 ± 0.63  1.83 ± 0.62  2.84 ± 0.72  1.89 ± 0.67  3.06 ± 0.74  1.73 ± 0.71 |

SD=Standard Deviation

**S2 Table. Dietary adherence among study participants.**

| **Outcomes variables** | **Mean ± SD** |
| --- | --- |
| Following BFG  F&V servings  Low GI  High sugar foods  High fiber foods  Carb spacing  n-3 FA  Healthy oils  High fat foods | 2.76 ± 2.13  1.85 ± 1.40  2.25 ± 1.45  1.42 ± 0.93  2.31 ± 1.50  2.87 ± 2.09  4.43 ± 2.03  1.36 ± 1.06  4.98 ± 2.06 |

SD=Standard Deviation; BFG=Benin Food Guide

**S3 Table. Cultural identity among study participants.**

| **Outcomes variables** | **Mean ± SD** |
| --- | --- |
| Spent time learning more about ethnic group.  Active in ethnic organizations or social groups  Clear sense of ethnic background  Think a lot about group membership.  Happy to be a member.  Have a strong sense of belonging.  Understand what group membership means.  Talked to others about group.  Have a lot of pride in ethnic group.  Participate in cultural practices.  Feel a strong attachment to group.  Feel good about cultural or ethnic background. | 2.71 ± 0.85  2.94 ± 0.75  2.99 ± 0.64  2.55 ± 0.72  3.23 ± 0.60  3.19 ± 0.63  3.06 ± 0.62  2.74 ± 0.78  3.27 ± 0.62  3.15 ± 0.62  3.17 ± 0.65  3.20 ± 0.63 |

**SD=Standard Deviation**
